# Actividad de fosfolipasas y proteasas en aislamientos de especies de *Candida* colonizadoras y causantes de vulvovaginitis en mujeres gestantes

**DOI:** 10.7705/biomedica.6759

**Published:** 2023-08-31

**Authors:** Martha Puello, Gregorio Young, Paola Suárez

**Affiliations:** 1 Grupo de Micología, Facultad de Medicina, Universidad de Cartagena, Cartagena, Colombia Universidad de Cartagena Grupo de Micología Facultad de Medicina Universidad de Cartagena Cartagena Colombia

**Keywords:** Candida, candidiasis vulvovaginal, fosfolipasas, endopeptidasas, factores de virulencia, microbiota, Candida, candidiasis, vulvovaginal, phospholipases, endopeptidases, virulence factors, microbiota

## Abstract

**Introducción.:**

Las proteasas y las fosfolipasas son factores de virulencia de *Candida* spp. que cumplen un papel importante en la invasión de los tejidos. Entre los factores relacionados con el huésped, se encuentran algunos asociados con las características ambientales y otros con la colonización.

**Objetivo.:**

Determinar la actividad de fosfolipasas y proteasas en aislamientos de especies colonizadoras y patógenas de *Candida* spp., aisladas de mujeres gestantes de Cartagena de Indias.

**Materiales y métodos.:**

Se determinó la actividad de fosfolipasas y proteasas en 56 aislamientos mediante degradación del sustrato y cálculo del coeficiente de actividad enzimática. Se compararon las actividades de fosfolipasas y proteasas, entre los aislamientos colonizadores y los patógenos.

**Resultados.:**

La actividad de la fosfolipasa fue "muy alta" (< 0,69) en 34 aislamientos e, igualmente, la de la proteasa en 14. No hubo diferencias significativas al comparar las actividades de las fosfolipasas y de las de las proteasas, entre los aislamientos colonizadores y los patógenos.

**Conclusiones.:**

La actividad de las fosfolipasas predominó como factor de virulencia en los aislamientos estudiados. No obstante, no se encontró una diferencia significativa entre los grupos de aislamientos colonizadores y los patógenos, en cuanto a las actividades de fosfolipasas y proteasas.

## Abstract (en)

**Introduction.:**

Proteases and phospholipases are virulence factors of *Candida* spp. that play an important role in tissue invasion. Among the factors related to the host some are associated with environmental characteristics and others with *Candida* colonization.

**Objectives.:**

To determine phospholipase and protease activities in colonizing and pathogenic strains, isolated from pregnant women in Cartagena de Indias.

**Materials and methods.:**

Phospholipase and protease activity was determined in 56 isolates, evaluating substrate degradation and calculating the enzyme activity coefficient. Phospholipase and protease activities were compared between colonizing and pathogenic strains.

**Results.:**

"Very high" (<0.69) phospholipase and protease activity was found in 34 and 14 isolates, respectively. There was no significant difference when comparing phospholipase and protease activities between colonizing and pathogenic isolates.

**Conclusions.:**

Phospholipase activity predominated as a virulence factor in the studied strains, but no significant difference was found between colonizing and pathogenic strains for phospholipase and protease activities.

El género *Candida* es parte de la microbiota humana, aunque puede producir enfermedad en los sujetos predispuestos que presenten algún grado de inmunosupresión ^1^. Estas levaduras son agentes causales comunes de infecciones invasoras y localizadas ^2^.

La candidiasis vulvovaginal es una infección que afecta especialmente a las mujeres en edad fértil, y se presenta al menos un episodio en el 75 % de las mujeres sanas ^3^. *Candida albicans* es responsable de la mayoría de los episodios de la candidiasis vulvovaginal, seguida por *C. tropicalis* o *C. glabrata*^3^^,^^4^.

Los factores de virulencia de *Candida* spp. y los relacionados con el huésped, son determinantes en el proceso de interacción entre ellos ^1^. Estos factores de virulencia incluyen enzimas hidrolíticas que les permite a los agentes patógenos adherirse, penetrar e invadir los tejidos ^1^. Entre estas hidrolasas están las proteasas que degradan diferentes tipos de proteína como el colágeno y la queratina. Las mejor caracterizadas son del tipo de las aspartil-proteasas, endopeptidasas cuyo nivel de expresión estaría asociado con un incremento de su virulencia y la exacerbación de síntomas ^1^. Otras enzimas son las fosfolipasas que hidrolizan los glicerofosfolípidos y están relacionadas con la invasión activa de los tejidos ^5^.

Por otro lado, entre los factores relacionados con el huésped se destaca el exceso de estrógenos en la vagina que, a su vez, determina la colonización por *Candida* spp., que sería el primer paso hacia la candidiasis vulvovaginal; este cambio aún no es muy bien entendido ^3^.

El presente estudio tuvo como objetivo determinar las actividades de las fosfolipasas y las proteasas en aislamientos colonizadores y patógenos de mujeres gestantes de Cartagena de Indias, con el fin de contribuir a la compresión de la candidiasis vulvovaginal.

## Materiales y métodos

Se determinó la actividad de las fosfolipasas y de las proteasas en 56 aislamientos provenientes de un estudio realizado en mujeres gestantes atendidas en la Clínica de Maternidad Rafael Calvo de Cartagena de junio a diciembre de 2014 ^3^. Los datos sociodemográficos de las 76 mujeres gestantes que participaron en el estudio se muestran en el cuadro 1. En el cuadro 2 se presentan los diagnósticos clínicos y de laboratorio realizados a las pacientes.

**Cuadro 1:** Datos sociodemográficos de las pacientes gestantes (n=76), portadoras de las cepas aisladas de *Candida* spp. El porcentaje faltante no respondió la pregunta.

Edad (años)	Origen %	Educación (años)* %	Ocupación %
Rango	Ciudad	>11	Amas de casa
16-36	(Cartagena)	10,4	90,8
	63,2		
Media	Departamental	11	Estudiantes
22,4 ± 6,07	(pueblos e islas)	46,1	3,9
	36,2		
Moda	No respondió	9-10	Profesionales
21	0,6	10,5	2,6
		<9	
Mediana		15,6	Otros
21		No respondió (17,4)	2,6

**Cuadro 2:** Frecuencia de diagnóstico médico frente a resultados de laboratorio en pacientes con diagnóstico de infección o colonización por *Candida* spp. (n=76)

Diagnóstico médico	n (%)	Resultados de laboratorio	n (%)
Candidiasis	22 (28,9)	Candidiasis	15 (19,7)
Vaginosis bacteriana	44 (57,9)	Vaginosis bacteriana	12 (15,8)
Candidiasis-tricomoniasis	1 (1,3)	Candidiasis - vaginosis bacteriana	1 (1,3)
Tricomoniasis	1 (1,3)	Infección bacteriana	1 (1,3)
No diagnosticada	8 (10,5)	Flora intermedia	40 (52,6)
		Microbiota normal	40 (52,6) 7 (9,2)

La clasificación de aislamientos colonizadores o patógenos se hizo de acuerdo con los criterios ya establecidos en un estudio previo ^3^. Los aislamientos conservados a -72 °C, se reactivaron en agar que contenía 4 % de Sabouraud (Merck, Alemania), a 37 °C por 24 horas.

Las cepas control utilizadas fueron: *Candida albicans* (ATCC 90028, referencia 0264 P, lote 264-19), *Candida tropicalis* (ATCC 750, referencia 0847 P, lote 84732-1), *Candida glabrata* (ATCC 64677, referencia 0226 P, lote 226-234), y *Candida guillermondii* (ATCC 6260, referencia R4601521 CL 1521, lote 1521).

Las actividades de fosfolipasas y proteasas se evaluaron por triplicado, según lo propuesto por Panizo *et al.*^6^, Ombrella *et al.*^7^ y Price *et al.*^8^. En el estudio para determinar la actividad de fosfolipasa, se utilizó un método semicuantitativo con yema de huevo; para la de proteasas, se usó un método semicuantitativo, utilizando agar de albúmina de suero bovino, para detectar la capacidad del microrganismo de producir la enzima.

Para preparar el inóculo, se recogieron de dos a tres colonias con ayuda de un asa estéril y se añadieron a 5 ml de solución salina estéril, hasta obtener una concentración de células equivalente a 0,5, según la escala de McFarland (aproximadamente 1-5 x 10^6^ UFC/ml).

Para la actividad de fosfolipasa, se inocularon 5 µl de la suspensión de la colonia en tres puntos equidistantes en una placa de 90 mm servida, aproximadamente, con 20 ml del siguiente medio: 65 g de agar Sabouraud glucosado; 58,45 g de cloruro de sodio; 0,0554 g de cloruro de calcio, 80 ml de yema de huevo y agua destilada hasta completar un litro. El pH del medio se ajustó a 6,3.

Todas las pruebas se practicaron por triplicado en días diferentes. Las placas se incubaron a 37 °C en aerobiosis. Las lecturas se hicieron a las 24, 48 y 72 horas ^2^^,^^3^ con un calibrador Vernier. Los cálculos de la actividad de fosfolipasas se hicieron de acuerdo con el método descrito por Price *et al.*^8^; el coeficiente de actividad enzimática (Pz) se determinó midiendo el diámetro de crecimiento de la colonia entre el diámetro de la zona considerada como indicadora de la producción de fosfolipasa . Se midieron en diferentes tiempos y se calculó el promedio de los resultados de los triplicados. Tal y como fue descrito para *C. albicans*^6^^,^^8^, se consideraron resultados negativos aquellos en los que el valor de Pz fue igual a 1 (actividad nula); resultados positivos con actividad débil, aquellos con Pz <1 y ≥0,64; y con actividad fuerte, aquellos con Pz <0,64 ^8^.

Este índice varía entre 0 y 1, y nos da una idea de la actividad enzimática de cada aislamiento. Los valores cercanos a 0 nos indican una máxima actividad de las fosfolipasas, mientras que valores próximos a 1, una baja actividad enzimática.

Para la actividad enzimática de las proteasas, se inocularon 5 µl de la suspensión de la cepa, preparada como se indicó previamente, en un medio que contiene: albúmina sérica bovina como única fuente de nitrógeno [0,5 g de fosfato dipotásico (Merck, Alemania)]; 0,04 g de sulfato de magnesio (Merck, Alemania); 1 g de cloruro de sodio (Merck, Alemania); 0,2 g de extracto de levadura (Oxoid, Reino Unido); 4 g de glucosa (Panreac, España); 0,5 g de albúmina sérica bovina (Merck, Alemania), y 4 g de agar-agar (Merck, Alemania), todo diluido en 200 ml de agua destilada ^6^^,^^7^.

Se calculó el coeficiente de actividad enzimática (Pz) como se describió anteriormente y se usó la escala descrita por Panizo *et al.* para medir la actividad fosfolipasa que se agrupa en cinco clases: negativa ^1^, débil (0,9 a 0,99), media (0,89 a 0,8), alta (0,79 a 0,7) y muy alta (menor de 0,69). Se compararon las actividades enzimáticas entre los aislamientos colonizadores y los patógenos, para lo cual se utilizó la prueba de ji al cuadrado, mediante el programa estadístico SPSS™, versión 25. Los valores de p menores de 0,05 se consideraron estadísticamente significativos.

## Resultados

En el estudio se observó que los aislamientos presentaron mayor actividad de fosfolipasas que de proteasas. Se encontró una actividad de fosfolipasa muy alta (cercana a 0) en 34 (60,71 %) de los aislamientos, alta en 5 (8,92 %), débil en 1 (1,78 %), media en ninguno y negativa (igual a uno) en 16 (28,57 %) del total (n=56) de aislamientos estudiados (n=56) de los cuales, 41 (73,21 %) correspondían a cepas colonizadoras y 15 (26,78 %) a cepas patógenas. En el cuadro 3 se muestran las frecuencias y porcentajes por especies.

**Cuadro 3:** Frecuencia y porcentaje de especies de *Candida* spp, colonizadoras y patógenas (n=56). Los cultivos fueron positivos en 54 pacientes, pero en dos muestras hubo crecimiento de dos especies.

Especies	Colonizadora (n=40)	Patógena (n=16)
	n (%)	n (%)
*Candida albicans*	24 (60)	10 (62,5)
*Candida tropicalis*	3 (7,5)	1 (6,3)
*Candida krusei*	2 (5,0)	1 (6,3)
*Candida parapsilosis*	2 (5,0)	1 (6,3)
*Candida dubliniensis*	1 (2,5)	1 (6,3)
*Candida glabrata*	2 (5,0)	0
*Candida lusitaniae*	1 (2,5)	0
*Candida norvegensis*	1 (2,5)	0
*Candida* spp.	4 (10)	2 (12,5)

Al comparar las actividades de fosfolipasas entre los aislamientos colonizadores y los patógenos, no hubo diferencia significativa en los dos grupos (p=0,671). Cuando se evaluó la actividad enzimática entre especies, se encontró que la actividad de las fosfolipasas fue muy alta en 27 aislamientos de *C. albicans* (cuadro 4).

**Cuadro 4:** Actividad de fosfolipasas de especies de *Candida* spp. en el total de aislamientos evaluados (n=56). Los valores altos son cercanos a cero y, los negativos, iguales a 1.

	Actividad de fosfolipasas					
	Negativa	Débil	Media	Alta	Muy alta	Total
Especie						
*Candida albicans*	2	1	0	4	27	34
*Candida dubliniensis*	1	0	0	0	0	1
*Candida tropicalis*	2	0	0	0	3	5
*Candida parapsilosis*	2	0	0	0	1	3
*Candida krusei*	1	0	0	1	1	3
*Candida glabrata*	2	0	0	0	0	2
*Candida lusitaniae*	1	0	0	0	0	1
*Candida norvengensis*	1	0	0	0	0	1
*Candida* spp.	4	0	0	0	2	6
Total	16	1	0	5	34	56

**Figura 1:**
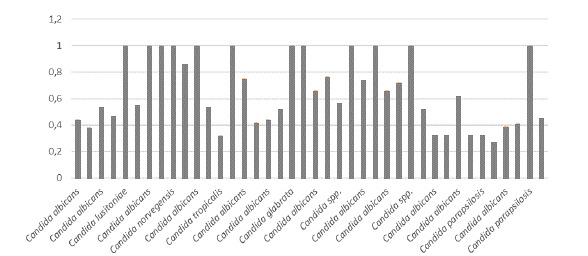
Actividad de fosfolipasas por especie de *Candida* spp. en los aislamientos colonizadores (muy alta < 0,69; alta = 0,79-0,70; media = 0,89-0,80; débil = 0,9-0,99; negativa = 1)

La actividad de proteasas fue negativa en 39 (69,64 %) cepas, débil en 1 (1,78 %), media en 1 (1,78 %), alta en 1 (1,78 %) y muy alta en 14 (25 %) del total de 56 aislamientos estudiados (n=56). No hubo diferencias significativas en la actividad de proteasas, entre los aislamientos colonizadores y los patógenos (p=0,366). Según las especies, la actividad de proteasas fue muy alta en 8 aislamientos de *C. albicans* (cuadro 5). Los resultados sobre la actividad de ambas enzimas en los aislamientos colonizadores y los patógenos, se muestran en las figuras 1 a 4.

**Cuadro 5:** Actividad de proteasas de especies de *Candida* spp. en todos los aislamientos evaluados (n=56). Los valores altos son cercanos a cero y los negativos, iguales a 1.

	Actividad de fosfolipasas					
	Negativa	Débil	Media	Alta	Muy alta	Total
Especie						
*Candida albicans*	23	1	1	1	8	34
*Candida dubliniensis*	1	0	0	0	0	1
*Candida tropicalis*	3	0	0	0	2	5
*Candida parapsilosis*	3	0	0	0	0	3
*Candida krusei*	2	0	0	0	1	3
*Candida glabrata*	2	0	0	0	0	2
*Candida lusitaniae*	0	0	0	0	1	1
*Candida norvengensis*	0	0	0	0	1	1
*Candida* spp.	5	0	0	0	1	6
Total	39	1	1	1	14	56

**Figura 2:**
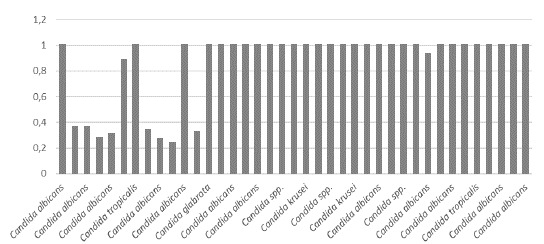
Actividad de proteasas por especie de *Candida* spp. en los aislamientos colonizadores (muy alta < 0,69; alta = 0,79-0,70; media = 0,89-0,80; débil = 0,9-0,99; negativa=1

**Figura 3:**
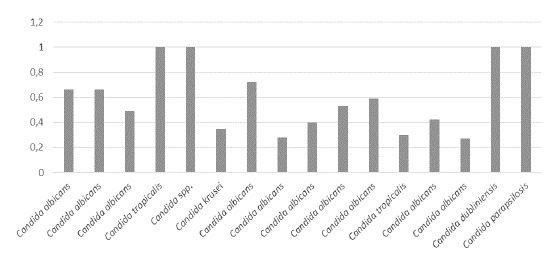
Actividad de fosfolipasas por especie de *Candida* spp. en aislamientos patógenos (muy alta <0 ,69; alta = 0,79-0,70; media = 0,89-0,80; débil = 0,9-0,99; negativa = 1)

**Figura 4:**
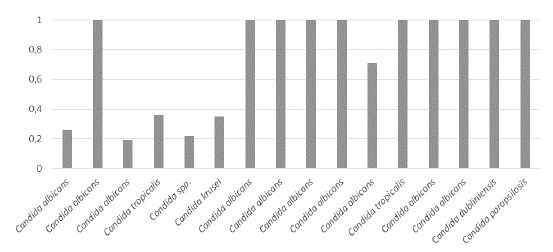
Actividad de proteasas por especie de *Candida* spp. en aislamientos patógenos (muy alta < 0,69; alta = 0,79-0,70; media = 0,89-0,80; débil = 0,9-0,99; negativa = 1)

## Discusión

La virulencia de las especies de *Candida* depende de diferentes factores, entre los cuales se destacan las enzimas hidrolíticas con actividades de esterasas, fosfolipasas, hemolíticas y proteolíticas, que facilitan la adhesión e invasión de los tejidos ^1^^,^^9^^,^^10^. La actividad de fosfolipasas se ha determinado en diferentes estudios de candidiasis vulvovaginal ^4^^,^^7^^,^^11^^,^^12^. Fule *et al.* solo encontraron actividad de fosfolipasa en *C. albicans* y no en especies no *albicans;* el 56,6 % de los aislamientos mostraron un coeficiente muy alto 4. En el presente estudio, se halló una actividad muy alta en 27 aislamientos de *C. albicans,* pero también, en aislamientos de *C. tropicalis, C. parapsilosis, C. krusei* y otras especies de *Candida.*

En el estudio de Shirkani *et al.* no se encontró correlación entre la actividad de fosfolipasa y la candidiasis vulvovaginal ^13^. En el presente estudio, tampoco se hallaron diferencias significativas entre los aislamientos de *Candida* colonizadores y los causantes de candidiasis vulvovaginal. En un estudio de Abu-Lubad en mujeres jordanas, se encontró una mayor actividad de fosfolipasas en *C. albicans*^12^, de la misma forma que se reveló en el presente estudio.

Son varios los estudios en los que se ha analizado la actividad de las proteasas en casos de candidiasis vulvovaginal ^6^^,^^7^^,^^11^^,^^12^. Por un lado, en el estudio ya mencionado de Fule *et al*., se halló una alta actividad de proteasa en todos los aislamientos, particularmente en los de C. *albicans* (97,1 %). En cuanto a la correlación con la candidiasis vulvovaginal, sí encontraron una asociación significativa con la actividad de proteasas ^4^. En el presente estudio, no se encontró una diferencia significativa entre los aislamientos colonizadores y los patógenos. Bassyouni *et al.* encontraron una alta actividad de proteasa en 82,5 % de los aislamientos de *C.* Asimismo, Abu-Lubad *et al.* encontraron que el 88,1 % de los aislamientos tenía una alta actividad de proteasa ^12^. En el presente estudio, solo el 25 % de los aislamientos estudiados mostró una muy alta actividad de proteasa, y esta se presentó en *C. albicans, C. tropicalis, C. krusei, C. lusitaniae* y *Candida* spp.

En conclusión, se observa un mayor porcentaje de la actividad de fosfolipasas como factor de virulencia en los aislamientos estudiados (60,71 %), pero no se encontró una diferencia significativa entre los grupos de aislamientos colonizadores y los patógenos. En los aislamientos estudiados, solo se detectó un 25 % de actividad de proteasas. Es probable que la actividad de fosfolipasa sea la que cumple un papel preponderante en el proceso de interacción entre huésped y parásito. Sin embargo, serían necesarios otros estudios que permitan una mejor comprensión de este proceso de patogénesis.
